# Why AI Monitoring Faces Resistance and What Healthcare Organizations Can Do About It: An Emotion-Based Perspective

**DOI:** 10.2196/51785

**Published:** 2025-01-31

**Authors:** Karl Werder, Lan Cao, Eun Hee Park, Balasubramaniam Ramesh

**Affiliations:** 1 Digital Business Innovation IT University of Copenhagen Copenhagen Denmark; 2 Information Technology & Decision Sciences Strome College of Business Old Dominion University Norfolk, VA United States; 3 Computer Information Systems J. Mack Robinson College of Business Georgia State University Atlanta, GA United States

**Keywords:** artificial intelligence, AI monitoring, emotion, resistance, health care

## Abstract

Continuous monitoring of patients’ health facilitated by artificial intelligence (AI) has enhanced the quality of health care, that is, the ability to access effective care. However, AI monitoring often encounters resistance to adoption by decision makers. Healthcare organizations frequently assume that the resistance stems from patients’ rational evaluation of the technology’s costs and benefits. Recent research challenges this assumption and suggests that the resistance to AI monitoring is influenced by the emotional experiences of patients and their surrogate decision makers. We develop a framework from an emotional perspective, provide important implications for healthcare organizations, and offer recommendations to help reduce resistance to AI monitoring.

## Introduction

Continuous monitoring through artificial intelligence (AI) technology is becoming increasingly important in health care. AI is becoming an integral part of health care, for example, in pain monitoring [1], in creating medical imaging platforms [2], and in delivering timely medical interventions to patients [3] to increase the quality of care, that is, the ability to access effective care [4]. AI monitoring solutions use machine learning techniques to learn from data generated from adhesive patches, sensor devices, video cameras, and other devices, and identify risks of illnesses and adverse events. Given the potential benefits that AI monitoring offers to health care [5], regulators, and healthcare (we use both terms, healthcare and health care. Healthcare refers to an industry or a system that provides people with health care, whereas health care refers to the process of care or things that health professionals do) organizations strongly advocate its use. For instance, the Food and Drug Administration has been actively approving remote monitoring devices for patient care [6], while prominent hospital systems like Stanford Medical are developing AI monitoring solutions for senior care [7].

However, novel solutions such as AI monitoring often encounter resistance from users [8,9]. Such resistance is usually attributed to users’ risk aversion toward innovation [10], sometimes called “liability of newness” [11], and their cognitive assessments of the costs and benefits of AI. However, we argue that emotions play an important role in AI monitoring resistance, even more than a rational evaluation of the decision, given that decision makers, specifically, patients and family members, lack extensive experience with novel AI solutions [10], which limits their ability to conduct a thorough cost-benefit analysis. Health care decisions often carry high stakes, and making the wrong choices can lead to serious consequences, including the loss of life. Therefore, making emotion-driven decisions in adopting AI-based solutions presents a significant challenge for healthcare organizations.

In this viewpoint paper, we discuss why the current view on resistance to AI monitoring may not fully capture the underlying reasons. We develop a framework from an emotional perspective that explains why decision makers resist AI monitoring and propose solutions that alleviate their concerns.

## What Is Missing in the Current View of AI Monitoring Resistance?

Healthcare organizations often attribute resistance to patients’ reluctance to accept innovative solutions to their cognitive assessment of the technology’s costs and benefits [12]. For instance, prior studies have reported that some patients’ resistance to AI systems results from their evaluations regarding privacy intrusion and insecurity of sensitive medical data because AI systems may be vulnerable to data breaches and data misuse [13]. Some patients may have doubts about the accuracy and reliability of AI systems [14] or do not understand and fully comprehend how AI functions, and hence, are reluctant to adopt these technologies [15]. Ethical dilemmas regarding AI decision-making in healthcare—in part, stemming from the fact that algorithms are inherently biased [16] with serious consequences of medical decisions—further contribute to the resistance of AI systems [13].

Taken together, the AI literature assumes that resistance decisions are rational and that patients themselves make the decisions about adopting the AI monitoring system. However, recent studies challenge these assumptions [8]. AI systems are opaque and often lack transparency [17], making it difficult for decision makers to rationally analyze their costs and benefits. Given the complexity of AI systems, decision makers often lack a comprehensive understanding of this advanced technology, exacerbating the challenges presented to rational decision-making [15]. Thus, we argue that alongside cognitive assessments of AI’s costs and benefits, emotions play a crucial role in AI monitoring resistance [3]. Decision makers may include others such as family members because, for example, senior citizens often have limited knowledge of technology and frequently turn to surrogate decision makers, such as their adult children, for help when making technology decisions [18,19].

## The AI Resistance Framework: An Emotion Perspective

Emotion is a complex psychological state that involves “loosely coupled changes in the domains of subjective experience, behavior, and peripheral physiology” [20]. Emotions are often triggered by external stimuli (eg, events or surrounding situations) [21], and they can be instrumental in directing attention to critical environmental details, refining decision-making, and aiding behavioral responses [22]. However, emotions can also be detrimental when they are inappropriate in type, intensity, or duration for a given situation. Therefore, individuals strive to regulate their emotions [22].

**Figure 1:**
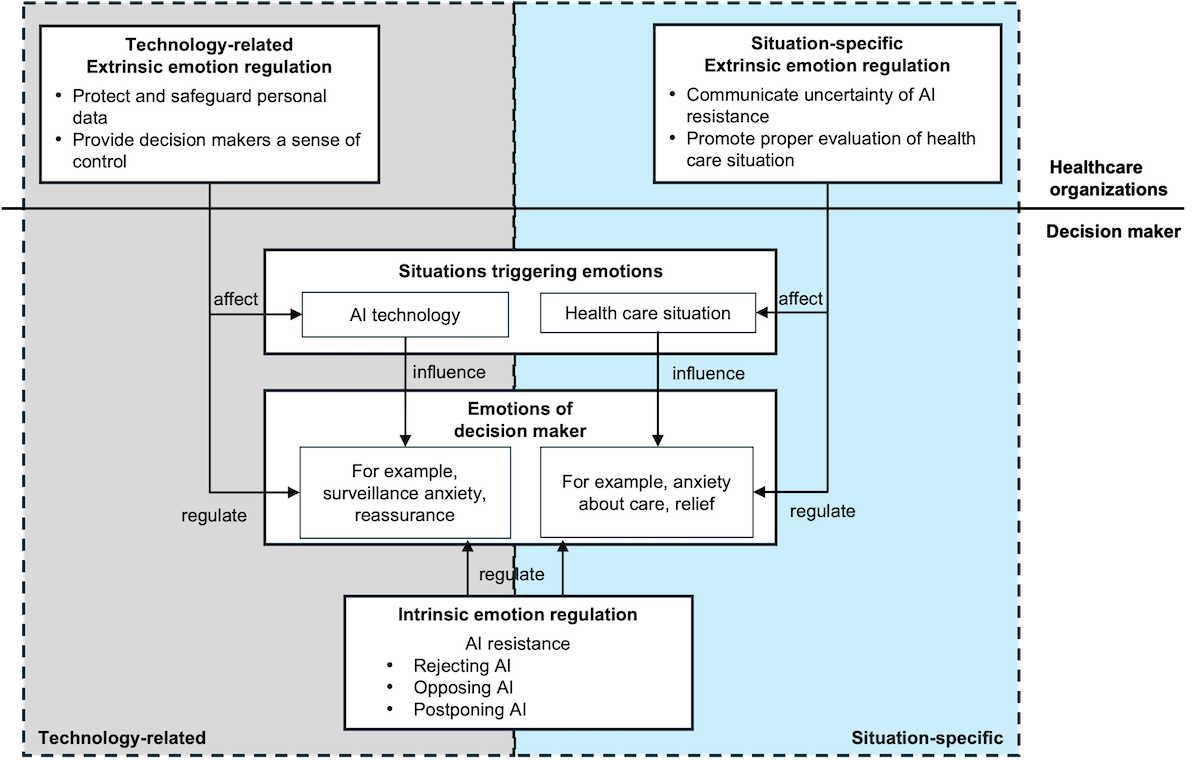
A framework of AI resistance from an emotion perspective. AI: artificial intelligence.

Emotion regulation refers to the “attempt to influence which emotions one has when one has them, and how one experiences or expresses these emotions” [20]. The goal of emotion regulation is to achieve some valued end (eg, decreasing negative emotion; Gross [20]). There are two types of emotion regulation [22]: Intrinsic emotion regulation focuses on regulating one’s own emotions, while extrinsic emotion regulation involves regulating another person’s emotions. According to a process model of emotion regulation proposed by Gross [23], there are five types of emotion regulation strategies: situation selection, situation modification, attentional deployment, cognitive change, and response modulation. These strategies can influence both the individuals practicing them and those around them [20]. This view on emotion regulation has attracted considerable interest across various domains, such as psychology, business, sociology, and healthcare, because of its potential to improve mental health, job performance, and social harmony, among others [20]. Focusing on the emotions experienced and regulated by decision makers, as well as policies and practices used by healthcare organizations that impact patients’ emotions, we present a framework that explains how emotions significantly impact resistance to AI monitoring (Figure 1).

## Decision Makers in Health Care

Health care is a complex process involving various stakeholders who may take on decision-making responsibilities based on a given situation. Patients are typically responsible for their own health decisions, such as following medical advice, using technological aids, or seeking further professional support. However, patients can also rely on others to guide them and sometimes even make important decisions on their behalf. For example, senior citizens often rely on family members for important medical and technology-related decisions. They may ask their adult children to install medical apps or help them navigate through their electronic healthcare records. Thus, key decision makers include patients and family members who act as surrogate decision makers [8,24].

## Emotions of Decision Makers and Their Triggers

### Overview

Emotions, both positive (eg, happiness and joy) and negative (eg, anxiety and fear), are triggered by specific experiences or events [25]. For example, decision makers may become anxious about adverse health outcomes. In the context of AI monitoring, emotions arise in response to the criticality of a given situation [26] and the capabilities of the AI solution [27]. Thus, we suggest that emotions play an important role in resistance to AI monitoring.

Figure 1 presents two noteworthy sources triggering negative emotions, such as anxiety or fear, and positive emotions, such as happiness and joy. The first source relates to AI technology. Emotions triggered by AI monitoring systems encompass both negative and positive emotions. Anxiety about patients’ surveillance and apprehension about transferring the responsibility of patient monitoring from the caretaker to the AI solution are common [8]. Conversely, positive emotions such as reassurance and comfort can arise from the continuous monitoring and alerts provided by AI systems, enhancing the sense of security for both patients and their families. The second source is specific to health care. Emotions triggered by the care process and its outcomes depend on the criticality of the health care situation. A critical health care situation, for example, would involve the possibility of the patient being hospitalized [8]. Conversely, positive emotions such as hope and relief can emerge when effective care processes and interventions are in place. In the following, we discuss some exemplary emotions triggered by both sources, AI technology and the health care situation.

### Emotions Triggered by AI Technology

AI technology can trigger a range of emotional responses. For example, reassurance is fostered by enhanced efficiency and reliability in patient care enabled by AI monitoring. With AI monitoring, specific care activities are managed by the system, which allows caretakers and health care providers to focus on more complex and personalized care tasks [28]. For example, AI monitoring can provide real-time alerts and updates about a patient’s condition, ensuring timely interventions and reducing the risk of human error. Decision makers feel reassured by the increased accuracy and efficiency that AI brings, as it supports health care providers in delivering high-quality care.

However, AI can also trigger negative emotions. Many decision makers are anxious because they do not know what information will be recorded. Surveillance anxiety is caused by sensor-based AI systems that provide extensive tracking of users and their behavior [27]. It is a feeling of tension and worry from thoughts resulting from the use of monitoring solutions. Many decision makers experience surveillance anxiety and worry about the continuous monitoring of patients and the collection and analysis of patient’s data, leading to privacy and possibly security concerns. Surprisingly, a recent study [8] has found that the level of health risk has a limited impact on surveillance anxiety. Rather, many worry about their patients being monitored even under conditions of high uncertainty, such as the high possibility of being hospitalized.

Delegation anxiety is caused by the delegation of some health care tasks to AI, leading to a loss of personal interaction between health care providers and patients [29]. With AI monitoring, certain care activities are delegated to the system, which reduces the workload of caretakers and care providers [28]. For example, AI monitoring can automatically communicate critical patient information to health care providers. Decision makers worry about a loss of personal interaction because technology use reduces the interaction between health care providers and their patients. Similar to surveillance anxiety, research has found that they experience delegation anxiety even when uncertainty is high, even though they are less likely to resist AI monitoring [8].

Technology can also provide positive emotional experiences. For example, when designing monitoring technology, providing rewards for goal attainment can reinforce self-efficacy and provide positive emotional responses that support a patient in managing their diabetes medication [30]. Reinforcements and reiterations of success are common techniques used in gamification, increasing positive emotional responses and motivation [31].

### Emotions Triggered by Health Care Situation

The health care situation of a patient also affects the emotions experienced by decision makers [8]. For example, anxiety about health care is experienced when decision makers worry about whether patients receive appropriate health care and monitoring of their health in highly uncertain situations. The process of care involves monitoring the health status [32] and providing physical, psychological, social, and spiritual support [33]. This was particularly acute during the pandemic, when decision makers were anxious about the prospect of their own or relatives’ health problems going unnoticed especially when they belonged to a high-risk population [34].

Anxiety about health outcomes is experienced when decision makers worry about the potential negative health outcome for the patient. The health outcome includes positive developments (eg, improvement of symptoms) and negative developments (eg, deterioration of symptoms and hospitalization) [35]. Anxiety about health outcomes varies with the level of uncertainty of health outcomes, that is, decision makers experience more anxiety about health outcomes when uncertainty is high and less when uncertainty is low.

Conversely, relief and hope can be experienced when decision makers are provided with sufficient care and treatment information. For example, knowing that patients receive continuous, attentive care and having access to clear, timely updates about their treatment progress can foster feelings of relief [36]. This information helps patients feel more secure about their health care situation and strengthens their trust in the health care received, enhancing their overall emotional comfort.

## Intrinsic Emotion Regulation With AI Resistance

Some emotions can be beneficial while others can be detrimental as they influence how decision makers perceive and interpret sensory information and shape their decisions based on such information, leading to either adaptive or maladaptive behaviors [20]. For example, decision makers’ emotions can increase and decrease resistance [8]. Resistance decreases with technology-induced relief, increases with technology-induced anxiety, and decreases with health care situation-specific anxiety. Since decision makers are motivated to regulate their own emotions, particularly negative emotions, such as anxiety, anger, and fear, it is crucial to guide them toward beneficial regulation activities [20].

To regulate emotions, decision makers can use an emotion regulation strategy called situation selection, where they take actions to increase or decrease the likelihood of being in a situation that is expected to elicit desirable or undesirable emotions [20]. For example, decision makers may postpone, reject, or oppose AI monitoring. Postponing the adoption of AI monitoring solutions can be a form of passive innovation resistance, allowing them to avoid the emotion-inducing situation altogether [37]. This strategy works when there is an alternative system in place and there is no mandate to move to the new system. Often, the decision to postpone is driven by the decision maker’s resistance to change as they feel more comfortable with the status quo.

However, decision makers often cannot delay a decision. Consequently, they may decide to reject AI monitoring due to a lack of prior experience. Decision makers’ resistance to change and satisfaction with the status quo can catalyze this, as well as contextual factors, such as functional or psychological barriers [37]. Functional barriers can occur when decision makers perceive substantial challenges or drawbacks associated with adopting an innovation, such as difficulties in use, perceived lack of added value, or potential risks. On the other hand, psychological barriers emerge when the innovation clashes with decision makers’ existing beliefs or perceptions, which may be influenced by sources like rumors or media [38]. Decision makers may oppose AI monitoring when they believe it is inherently unsuitable for their needs, even prior to a thorough evaluation. This resistance can manifest as attacking AI monitoring and the spreading of negative opinions about it [39].

## Extrinsic Emotion Regulation Through Managerial Actions

### Overview

Healthcare organizations and governmental institutions, as well as external entities with significant influence, have a vested interest in regulating decision makers’ emotions. As collective actors, these organizations engage in cognitive reasoning and shape emotional understanding to guide thinking and actions [40,41]. They also practice collective emotion regulation to ensure informed decision-making [42]. In addition to regulating the emotions of their collective members, these organizations can also engage in extrinsic emotion regulation. This involves managing the emotions of external individuals, such as decision makers including patients and their families, to mitigate the rejection of AI innovations given their benefits and performance gains. We suggest the following emotion regulation strategies for organizations to manage decision makers’ emotions and mitigate AI resistance.

### Protect and Safeguard Personal Data for Responsible AI Solutions

Organizations can manage decision makers’ emotions by adopting the emotion regulation strategy called situation modification. This involves actively changing situations and reshaping events that induce negative emotions to reduce their effects [20]. More precisely, organizations need to be cognizant of decision makers’ emotions that may arise from a specific health care situation and the use of AI monitoring. A significant technology-related emotion is anxiety about the unauthorized disclosure and use of personal data. Healthcare organizations should carefully evaluate and choose an AI monitoring solution that is designed to protect and safeguard personal and other sensitive data collected from decision makers and others involved in a patient’s care, for example, by blurring images or deidentifying the data before they are stored [8]. As AI monitoring solutions collect, store, and process data to provide predictions and recommendations, organizations need to balance ethics, personal privacy, and data security considerations against gains in quality of care and health outcomes [43]. In addition, healthcare organizations should make sure that there are sufficient human interactions with patients when deploying AI monitoring solutions. Healthcare organizations should make decision makers aware that AI monitoring can even be used to enhance engagement by enforcing personal interactions and providing personalized care.

Governmental institutions should proactively establish proper laws and regulations to manage AI monitoring. Examples include a blueprint from the White House that seeks to prevent negative implications of AI and its monitoring [44] and a draft from the European Union that seeks to ban AI for mass monitoring [45]. These initiatives are in response to criticism about some AI monitoring practices [46]. Seemingly, organizations are often too lax when managing personal data. However, when organizations take these concerns seriously, they can develop and deploy responsible AI solutions [47]. For instance, increasing transparency of essential algorithms, their data, and data processing allows others to evaluate the appropriateness of the AI solution and engages them in an open debate about corporate and governmental responsibilities concerning the design and deployment of AI solutions.

### Provide Decision Makers a Sense of Control

Organizations can also manage decision makers’ emotions by giving them a sense of control [8], that is, the feeling of being empowered and having the capability to influence or manage the operation of AI systems and their impact on decision makers’ personal or professional lives. Decision makers typically regulate their emotions by choosing situations that either enhance or reduce the chances of experiencing favorable or unfavorable emotions [20].

Here, organizations can support decision makers by creating environments where they experience this sense of control. For instance, healthcare organizations should ensure that AI solutions are designed to provide decision makers with options to change the schedule, frequency, and type of data collected. Providing sufficient choice, freedom, and autonomy regarding what is monitored can alleviate decision makers concerns and reduce their negative technology-induced emotions, such as surveillance anxiety [8].

Additionally, regular training and educational resources on AI systems can help decision makers understand and confidently engage with these tools. These resources should cover how AI operates, the available options, and the benefits and limitations of the technology. By fostering a thorough understanding, organizations can help decision makers feel more in control and less anxious.

### Communicate the Risk of AI Resistance

Healthcare organizations should communicate the potential consequences of not using AI monitoring to address resistance and foster adoption. First, healthcare organizations need to communicate the risk with the status quo. Health risks might already be present and decision makers seek to manage such risks. Many decision makers may even lack a clear understanding of the risks faced by the patients. For example, more than one out of four people aged 65 years or older falls each year [48], but less than half tell their doctor and caretaker [49] about these incidents. Therefore, it is important to communicate to decision makers the risk of adverse health outcomes such as a fall that may go undetected in the absence of adequate monitoring.

Second, healthcare organizations need to communicate the risk of AI resistance, that is, they need to articulate how AI capabilities can alleviate existing risks through its innate ability to act. For example, in addition to educating the decision makers about the ability of AI monitoring systems to continuously monitor potential symptoms and provide early detection of serious illnesses [50], healthcare organizations can ask decision makers to think about what would happen if patients’ abnormal activities and adverse events such as falls are not noticed on time. When healthcare organizations also communicate the risk of AI resistance, decision makers can assess the existing risks more comprehensively, and thus, may choose to adopt AI monitoring.

### Promote Proper Evaluation of Health Care Situation

Despite the threats and challenges presented by AI, the technology offers significant advantages, such as assistance in identifying health problems, monitoring for adverse events (eg, falls), and detecting abnormal behaviors (eg, wandering). When decision makers focus on these advantages and the opportunities AI provides in a given health care situation, they experience positive emotions such as reassurance and are more likely to evaluate the systems in a positive light. To assist decision makers in navigating healthcare processes and associated AI tools, organizations should enhance communication by providing clear, consistent updates about AI-powered healthcare procedures, patient status, and quality measures [51,52]. This transparency helps reduce concerns and anxiety, ensuring decision makers feel informed and confident in the AI systems. They should also develop personalized care plans that integrate AI system capabilities to address each patient’s unique needs and involve family members in the planning process [53]. Tailoring care with AI support fosters trust and reassures decision makers by demonstrating a commitment to their needs.

In addition, establishing robust crisis management protocols that incorporate AI tools for real-time monitoring and response provides immediate support and reassurance [54]. This helps decision makers feel secure about the organization’s ability to manage emergencies effectively. By implementing these strategies, healthcare organizations can mitigate AI resistance by enhancing trust, reducing anxiety, and fostering a positive emotional response toward AI-assisted care.

## Abbreviations

AI: artificial intelligence
